# Preservation of retinal structure and function in two mouse models of inherited retinal degeneration by ONL1204, an inhibitor of the Fas receptor

**DOI:** 10.1038/s41419-024-06970-6

**Published:** 2024-08-08

**Authors:** Mengling Yang, Jingyu Yao, Lin Jia, Andrew J. Kocab, David N. Zacks

**Affiliations:** 1 Department of Ophthalmology and Visual Sciences, University of Michigan, Kellogg Eye Center, Ann Arbor, MI USA; 2 Eye Center of Xiangya Hospital, Xiangya School of medicine, Central South University, Changsha, Hunan China; 3 ONL Therapeutics, Inc., Ann Arbor, MI USA

**Keywords:** Target validation, Peptide delivery

## Abstract

Due to the large number of genes and mutations that result in inherited retinal degenerations (IRD), there has been a paucity of therapeutic options for these patients. There is a large unmet need for therapeutic approaches targeting shared pathophysiologic pathways in a mutation-independent manner. The Fas receptor is a major activator and regulator of retinal cell death and inflammation in a variety of ocular diseases. We previously reported the activation of Fas-mediated photoreceptor (PR) cell death in two different IRD mouse models, rd10 and P23H, and demonstrated the protective effect of genetic Fas inhibition. The purpose of this study was to examine the effects of pharmacologic inhibition of Fas in these two models by intravitreal injection with a small peptide inhibitor of the Fas receptor, ONL1204. A single intravitreal injection of ONL1204 was given to one eye of rd10 mice at P14. Two intravitreal injections of ONL1204 were given to the P23H mice, once at P14 and again at 2-months of age. The fellow eyes were injected with vehicle alone. Fas activation, rate of PR cell death, retinal function, and the activation of immune cells in the retina were evaluated. In both rd10 and P23H mice, ONL1204 treatment resulted in decreased number of TUNEL (+) PRs, decreased caspase 8 activity, enhanced photoreceptor cell counts, and improved visual function compared with vehicle treated fellow eyes. Treatment with ONL1204 also reduced immune cell activation in the retinas of both rd10 and P23H mice. The protective effect of pharmacologic inhibition of Fas by ONL1204 in two distinct mouse models of retinal degeneration suggests that targeting this common pathophysiologic mechanism of cell death and inflammation represents a potential therapeutic approach to preserve the retina in patients with IRD, regardless of the genetic underpinning.

## Introduction

Inherited retinal degeneration (IRD) encompasses a group of retinal disorders arising from mutations in ~300 distinct genes [1]. The considerable genetic diversity underlying IRD has impeded the creation of therapies targeted at specific mutations [2]. While mutation-specific treatments could potentially be viewed as the ideal [3], the prospect of developing such therapies for each of the nearly 300 implicated genes presents a daunting challenge in terms of both cost and time. Consequently, there exists a significant unmet medical need for therapeutic approaches that target commonly shared pathophysiological pathways that contribute to retinal degeneration. This approach could allow for a mutation-independent therapies for patients with IRD.

A promising target for promoting photoreceptor (PR) protection is the cell surface receptor Fas (CD95), which plays a central role in activating cell death and in fostering the inflammatory microenvironment associated with retinal diseases [4–6]. Extensive work in pre-clinical models of diseases such as retinal detachment [7–10], age-related macular degeneration (AMD) [11, 12], and glaucoma [6, 13] has shown that Fas is highly upregulated in these conditions and that inhibiting the activation of the Fas receptor leads to retinal protection. This is achieved through both the direct suppression of the cell death pathway and by reducing the inflammation response induced by the disease [6, 8, 11–16]. These findings indicate that despite the varying specific stressors in each disease, the pathologic response leading to degeneration is commonly mediated by the Fas receptor. Based on this, we hypothesize that Fas also contributes to PR degeneration in IRD and that targeting Fas receptor activity could offer a mutation-independent strategy for retinal preservation.

We previously reported the activation of Fas-mediated PR cell death in two different mouse models of IRD, rd10, and P23H, demonstrated by elevated mRNA levels of Fas and increased staining for Fas receptor in the retinas of both strains [17]. In P23H mice, as in the human condition, a specific mutation causes the replacement of proline with histidine at the 23rd amino acid position of the rhodopsin (RHO) protein [18, 19]. The P23H mutation is a common cause of autosomal dominant retinitis pigmentosa, with the amino acid substitution leading to misfolding of the RHO protein [20–23], increased endoplasmic reticulum (ER) stress, and the activation of cell death pathways including apoptosis, necroptosis, and autophagy [24–35]. The rd10 mouse model is characterized by a missense mutation in the gene encoding the β subunit of cyclic guanosine monophosphate (cGMP) phosphodiesterase 6 (PDE6). This mutation results in continuous accumulation of cGMP, leading to excessive calcium influx, oxidative stress, inflammation, and subsequent death of PR cells [36–39]. In humans, loss-of-function mutations in the Pde6-β gene are associated with autosomal recessive retinitis pigmentosa. Notably, around 5% of retinitis pigmentosa (RP) cases are attributed to mutations in the gene encoding PDE6-β [40, 41]. We recently published a proof-of-concept study that demonstrated the protective effect of genetic inhibition of the Fas receptor in these two mouse models of IRD [17].

In this study, we expand on that work to examine the effects of pharmacologic inhibition of Fas in these two models by intravitreal injection of a small peptide inhibitor of the Fas receptor, ONL1204. Our peptide is based on studies by Zou and colleagues, who found that a YLGA amino-acid motif located near the extracellular N terminus of the alpha-subunit of the Met oncogene is necessary and sufficient to specifically bind the extracellular portion of Fas and to act as a potent FasL antagonist and inhibitor of Fas trimerization in a mouse model of fatty liver disease [42]. In our previous studies, we have shown that in both cell culture and retinal detachment model, treatment by a small peptide Met12, a precursor molecule to ONL1204, has significant protective effects in mouse models of PR degeneration caused by retinal detachment or AMD [7, 12]. This protective effect has been further validated with a derivative of Met12, named ONL1204, in a mouse model of glaucoma [13]. ONL1204 is currently used in human clinical trials [11]. In this study, we observed that intravitreal administration of ONL1204 in both P23H and rd10 mice led to a decrease in Fas activation, a reduction in inflammatory response, and the preservation of PR structure and function.

## Materials and methods

### Animals and intravitreal injection

The animal experiments adhered to the ethical guidelines set forth by the Association for Research in Vision and Ophthalmology (ARVO) for the use of animals in ophthalmic and visual research and were approved by the institutional Animal Care & Use Committee at the University of Michigan. P23H (strain #017628), rd10 (strain # 004297), and C57BL/6J mice were purchase from Jackson lab. For experiments with P23H, only heterozygous were used, as this represents the more common clinical presentation. Mice were housed in a 12-h light-dark at the University of Michigan Kellogg Eye Center vivarium, with a cage light intensity maintained at 300 lux.

ONL1204, in a volume of 1 µL at 2 mg/ml, 2 µg per eye, was administered via intravitreal injection to one eye of the mice, and the fellow eyes were injected with vehicle solution as controls. This dose of ONL1204 has been shown to be effective in other ocular disease mouse models [11, 13]. In P23H mice, two intravitreal injections were given, once at P14 and again at 2-months of age. In rd10 mice, a single intravitreal injection was given at P14. We observed partial cataracts on some mice across all groups, which may be induced by the injection procedure, especially in P23H groups that received two injections. All data was included in the analysis without removing the animal with partial cataracts. Since we were comparing the data of ONL1204 treated eye to vehicle treated fellow eye of each mouse, the study was not double-blinded/randomized. The ONL1204 sequence is published in the patent # WO 2016/178993 Al and is available on the NIH-PubChem website.

### Antibodies

Primary antibodies: RHO (4D2, Novus Biologicals, NBP1-48334; 1:2000), m-Opsin (Millipore, AB-5405,1:1000), Iba1 (1:100, Novus Biologicals, NB100-1028, Littleton, CO, USA); Secondary antibodies are from Invitrogen (Paisley, UK): goat anti-mouse Alexa Fluor 546 (1: 1000, A-11030), goat anti-rabbit Alex Fluor 488 (1: 1000, A-10008) and donkey anti goat Alexa Fluor 488 (1: 1000, A-11055).

### Histology

Eyes were carefully extracted after marking the corneal 12 o’clock position to maintain orientation. Eyes were then fixed in 4% paraformaldehyde overnight at 4 °C. Subsequently, these specimens were embedded in paraffin and sectioned into 6 µm sections using a Shandon AS325 microtome (Thermo Scientific, Cheshire, England, UK). Hematoxylin and eosin staining, acquired from Fisher Scientific (Hercules, CA, USA), was conducted as described previously [43]. We selected for staining only those sections crossing the optic nerve, encompassing both the superior and inferior regions of the retina. Images of these sections from comparable areas was captured utilizing a Leica DM6000 microscope (Leica Corp., Wetzlar, Germany).

### Immunofluorescence on retinal sections

Retinal sections crossing the optic nerve were blocked in 5% goat serum in PBS with 0.1% Triton X-100 (PBST; Sigma-Aldrich) for an hour, then rinsed for three times with PBST. After overnight incubation at 4 °C with primary antibodies, sections were washed with PBST and incubated with secondary antibodies for 1 h at room temperature. Slides were then mounted using ProLong Gold with DAPI (Invitrogen, P36941). Images were taken at the comparable retina area (600 µm from the optic nerve) using a confocal microscope (Leica STELLARIS 8 FALCON Confocal Microscope) at a fixed gain. In this study, staining of RHO and m-Opsin was performed on 10 µm sections, whereas Iba1 staining utilized 30 µm sections. (*n* = 8 mice for P23H; *n* = 8 mice for rd10).

### Immunofluorescence on retinal whole mount

Retinas were prepared following a method previously detailed [12]. Retinal samples underwent a 48-h incubation with Iba1 antibody at 4 °C. Post-incubation and washes, the retinas were subjected to secondary antibodies overnight at 4 °C followed by three additional washes. Retinas were then incubated with DAPI at room temperature for 2 h and subsequently positioned on a glass slide (Fisher Scientific, Pittsburgh, PA, USA) with the ganglion cell layer oriented upwards. Using a Leica STELLARIS 8 FALCON Confocal Microscope, Z-section confocal imagery was obtained from matching areas of superior and inferior regions of the retina. (*n* = 7 mice for P23H; *n* = 8 mice for rd10).

### Terminal deoxynucleotidyl transferase dUTP nick end labeling staining

Terminal deoxynucleotidyl transferase dUTP nick end labeling (TUNEL) was applied to 10 µm cryo sections intersecting the optic nerve. The eyes of P23H were analyzed at 1 month. Meanwhile, those from rd10 were examined at P28. The DeadEnd Colorimetric TUNEL System (Promega Corporation, Madison, WI, USA) was employed as per the manufacturer’s guidelines. For the P23H samples, 4 non-overlapping images cross optic nerve were captured at 20× magnification. The aggregate of TUNEL-positive photoreceptors across the entire section was enumerated and averaged for individual samples. For the rd10 samples, four 40× magnification images were acquired at intervals of 500 and 1000 µm, both above and below the optic nerve. The tally of TUNEL-positive photoreceptors within each 40× magnification image was computed and averaged. (*n* = 13 mice for P23H; *n* = 13 mice for rd10).

### Caspase 8 activity assay

Protein extraction, intended for identifying caspase 8 activity, adhered to a method detailed earlier [17]. Two to three retinas of left eyes (ONL1204 injected) or right eyes (control vehicle injected) from 2 to 3 mice were pooled as one sample, then homogenized in a lysis buffer (20 mM MOPS, pH 7.0, 2 mM EGTA, 5 mM EDTA, and 0.1% Triton X-100) with the presence of protease inhibitor (complete protease inhibitor tablet, 11697498001, Roche, Indianapolis, IN, USA). Samples were centrifuge at 10,000 × *g* for a 1 min at 4 °C. Subsequently, each assay utilized 150 µg of protein. Samples were loaded in duplicate and Caspase 8 activity was analyzed using the Caspase-8 Activity Assay Kit (Colorimetric) (NBP2-54817, Novus Biologicals) with a plate reader (Agilent BioTek Gen-5, Santa Clara, CA 95051). The values of caspase 8 activity were then normalized to the ONL thickness of the retina as measured by OCT. (*n* = 7 mice for P23H; *n* = 10 mice for rd10).

### Optical coherence tomography

Optical coherence tomography (OCT) was executed following a previously outlined methodology [42] with the spectral domain OCT equipment provided by Bioptigen, Inc. (Durham, NC, USA). For P23H, the outer nuclear layer (ONL) thickness was gauged from both superior and inferior of the retina at distances of 250 and 500 µm from the optic nerve. For the rd10 mice, measurements were taken at a 500 µm distance from the optic nerve in the superior, inferior, temporal, and nasal retinal regions. (*n* = 20 mice for P23H at 3 months of age; *n* = 16 mice for P23H at 4 months of age; *n* = 21 mice for rd10 at P28; *n* = 16 mice for rd10 at P35; *n* = 12 mice for rd10 at P42).

### Electroretinography

Electroretinography (ERG) assessments were conducted with the Espion E2 system (Diagnosys, Lowell, MA, USA), following a method delineated earlier [27]. Mice were dark adapted for overnight, scotopic ERG readings were obtained at 0.01, 1, 10, 32, and 64 log cd s/m^2^. After a 10-min light-adaptation period, photopic response was recorded at intensities of 10, 32, and 100 log cd s/m^2^. The amplitudes were then measured through the Espion V6 software suite. (*n* = 20 mice for P23H at 3 months of age; *n* = 13 mice for P23H at 4 months of age; *n* = 10 mice for rd10).

### Statistical analysis

To compare ONL1204-injected eyes with those injected with Vehicle, a one-tailed Wilcoxon test was utilized. For evaluations among 3 groups, a 1-way ANOVA was utilized, followed by a Tukey multiple comparison test. The statistical evaluations and charting were facilitated using Prism (GraphPad, Inc., La Jolla, CA, USA) and Microsoft Office Excel (Richmond, WA, USA). Data is conveyed as mean ± standard deviation, with distinctions deemed notable when *P* < 0.05.

## Results

### Intravitreal injection of ONL1204 in the P23H mouse

**Fig. 1:**
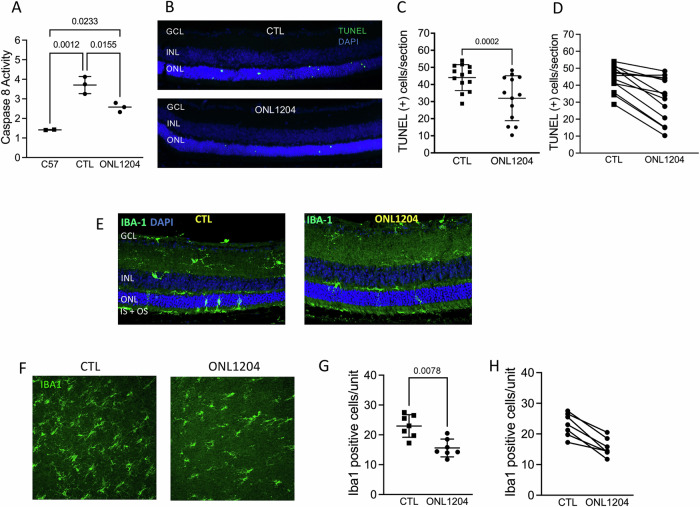
ONL1204 injection reduced Fas-mediated photoreceptor cell death and immune cell activation in the P23H retina. **A** Quantification for caspase-8 activity in the retina of ONL1204 and vehicle injected (CTL) P23H mice at 1 month, normalized to age matched C57 mice. (Pooled 2–3 retinas for each sample; 7 mice for P23H, 4 mice for C57). **B** Representative TUNEL staining images and (**C**) quantification of TUNEL-positive cells in the ONL of vehicle and ONL1204 injected P23H at 1 month of age (*n* = 13 mice). **D** Paired line graph represents the value of ONL1204 and control vehicle injected eye from each mouse. **E** Representative immunostaining images of retinal sections from inferior retinas of vehicle and ONL1204 injected P23H mice at 1 month of age stained with Iba-1 and DAPI. **F** Representative images of the outer nuclear layer from inferior area of the retinal whole mount of ONL1204 and vehicle injected P23H mice stained with Iba1 at 1 month of age. **G** Quantification of Iba1-positive cells in the ONL and subretinal space of the inferior retina of vehicle-injected P23H and ONL1204-injected P23H mice (*n* = 7 mice). **H** paired line graph shows the value of ONL1204, and control vehicle injected eye from each individual mouse. one-tailed Wilcoxon test. GCL ganglion cell layer, INL inner nuclear layer, ONL outer nuclear layer, IS inner segment,OS outer segment.

Our previous works have shown that in mouse models of retinal detachment and AMD, treatment by Met12, a precursor molecule to INL1204, inhibited Fas-mediated caspase 8 activation [7, 12], and reduced activities of caspase 3 and caspase 9 as well [7]. This protective effect was further validated with ONL1204 in a mouse model of glaucoma [13]. In this study, caspase 8 activity assay was used as a readout of Fas-dependent caspase activation. Given the relatively slower degeneration of the P23H mouse retina, we performed two intravitreal injections of ONL1204, the first at postnatal day 14 (P14), when the eyelids of the mice first open, and the second at 2 months of age. The fellow eyes were injected with vehicle alone. Caspase 8 activity, an early marker of the activated Fas receptor, was measured 2 weeks after the first injection. Normalized caspase 8 activity in retinas of ONL1204 injected eyes was significantly lowered as compared to vehicle injected eyes (Fig. 1A), indicating a reduction in Fas activation. This was associated with a significant decrease in TUNEL staining in the ONL of the retina as compared to vehicle-injected control eyes (Fig. 1B–D).

In order to evaluate the impact of ONL1204 injection on immune activation in P23H mice, we collected retinal samples from both ONL1204 injected eyes and control vehicle injected fellow eyes for subsequent Iba1 immunostaining on both 30 μm sections and on retinal whole mount. Compared to control vehicle treated eyes, a notably reduced number of Iba1-positive cells were observed within the ONL of the retinal sections of ONL1204 treated mice (Fig. 1E) as well as in the retinal whole mount (Fig. 1F–H). These results are consistent with our previous work showing that Fas knockout reduced immune cell activation and migration within P23H retinas [17].

**Fig. 2:**
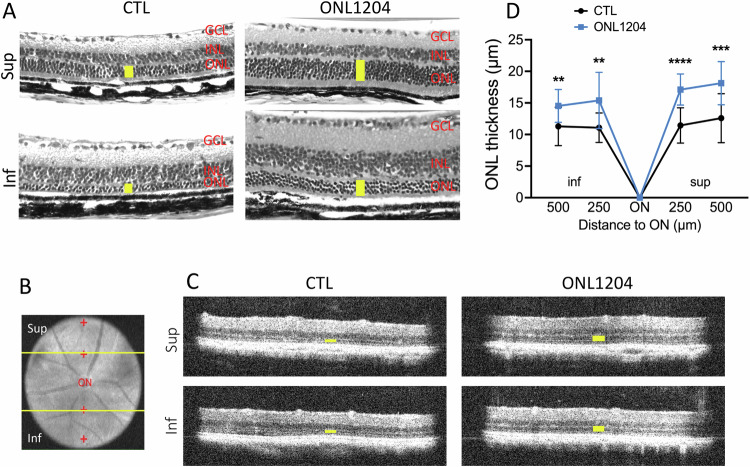
Increased photoreceptor survival in ONL1204 injected P23H retina compared with vehicle injected control eyes. **A** Representative H&E staining images show preserved photoreceptor layer (yellow bars indicate the ONL) in the retina of ONL1204 injected eyes compared with control vehicle injected eyes at 4 months of age. **B** Fundus image demonstrates the representative OCT images in (**C**) were extracted from the comparable area from both superior and inferior of the retina. **C** Representative OCT images of superior (sup) and inferior (inf) retina of 4-month-old ONL1204-injected P23H and vehicle-injected P23H control. **D** Quantification of the thickness of the ONL (indicated by yellow bars in the OCT images) of the superior and inferior retina measured at both 250 and 500 µm from the optic nerve head by OCT in ONL1204 and control vehicle injected P23H mice at the age of 4 months (*n* = 16 mice). **p < 0.01; ***p < 0.001; ****p < 0.0001. one-tailed Wilcoxon test. GCL ganglion cell layer, H&E hematoxylin-eosin, INL inner nuclear layer, ONL outer nuclear layer, IS inner segment, OS outer segment, Sup superior, Inf inferior.

In P23H mice, photoreceptor degradation is more pronounced in the inferior retina as compared to the superior portion of the retina [33]. As such, we analyze sections in both superior and inferior portions of the retina. Histological analysis of retinal samples at 4 months of age, after two injections of ONL1204, showed a significant thinning of the ONL in the vehicle treated eyes, whereas ONL1204-treated eyes maintained a thicker ONL (Fig. 2A). The thickness of the ONL which consists of the nuclei of the photoreceptor cells, was assessed in vivo by OCT in both the superior and inferior retina (Fig. 2B). Injection of ONL1204 resulted in significantly thicker ONL in both the superior and inferior portions of the retina compared with their vehicle injected fellow eyes at age of 3 and 4 months (Fig. 2C, D).

**Fig. 3:**
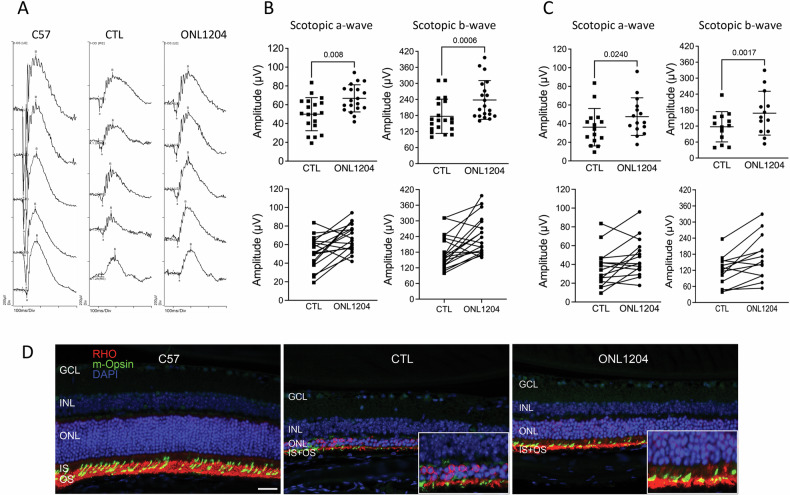
Preserved retinal function in the eyes of ONL1204 injected P23H mice. **A** Representative scotopic (at 0.01, 1, 10, 32 and 64 cd s/m^2^) ERG at 3 months of age in ONL1204-injected P23H, vehicle-injected P23H, and C57 mice. **B** Quantification of amplitudes of scotopic a-wave and scotopic b-wave (32 cd s/m^2^) at 3 months and (**C**) 4 months of age confirms the preservation in retinal function. Line graph shows the paired value of both eyes from each individual mouse. (*n* = 20 mice for 3 months of age, *n* = 13 mice for 4 months of age); **D** Representative immunostaining images of inferior retina of four-month-old C57 and injected P23H mice, stained with rhodopsin (RHO in red), m-opsin (green), and DAPI (blue). The white rectangular inserts are zoomed-in areas highlighting the staining in the inner and outer segments of the retinas. one-tailed Wilcoxon test. INL inner nuclear layer, ONL outer nuclear layer, IS inner segment, OS outer segment. Scale bar: 25 μm.

The functions of the photoreceptor and the retina were assessed by ERG recording at 3- and 4-months of age. We also performed immunostaining of the photoreceptor proteins, rhodopsin and cone m-opsin. Visual function of rod photoreceptors measured by both a-wave and b-wave of scotopic ERG showed significantly higher responses in ONL1204-treated eyes compared to their vehicle-treated fellow eyes (Fig. 3A–C). Consistent with our finding in the genetic knockout of Fas function in the P23H mouse [17], the ONL1204 treated eyes were still well below the ERG responses observed in wild type C57 mice. The improved ERG responses in ONL1204-treated eyes, as compared to vehicle-treated eyes, were correlated with increased staining for both rhodopsin and m-opsin in the retinas of P23H mice injected with ONL1204 as compared to the control vehicle injected eyes (Fig. 3D).

### Intravitreal injection of ONL1204 in the rd10 mouse

**Fig. 4:**
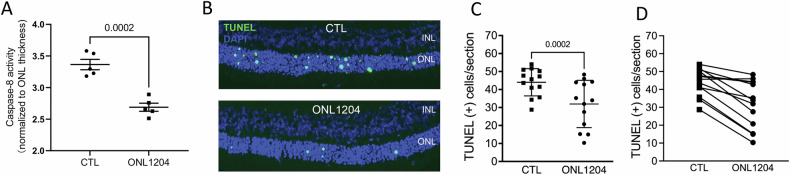
Intravitreal injection of ONL1204 reduced photoreceptor cell death in the rd10 retina. **A** Quantification of caspase 8 activity in the pooled retinas of vehicle and ONL1204 injected eyes of rd10 mice at P21. (Pooled 2 retinas for each sample; 10 mice). **B** Representative TUNEL staining images and (**C**) quantification of TUNEL-positive cells in the ONL for control vehicle and ONL1204 injected rd10 mice at P21. **D** Line graph shows paired value of both eyes from each individual mouse. (*n* = 13 mice), one-tailed Wilcoxon test. INL inner nuclear layer, ONL outer nuclear layer.

**Fig. 5:**
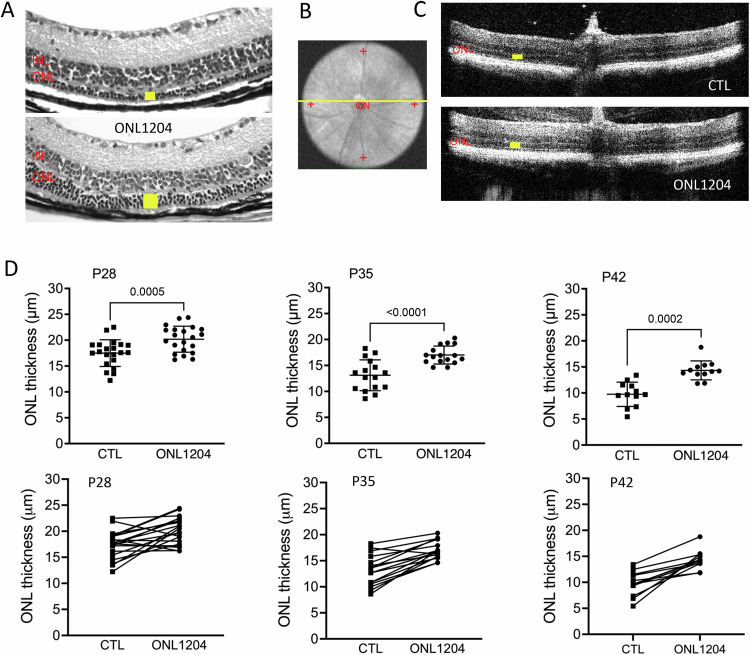
Increased photoreceptor survival in ONL1204 injected eyes of rd10 mice compared with vehicle injected eyes. **A** Representative H&E staining images show preserved photoreceptors (yellow bars indicated ONL) in the retina of ONL1204-injected rd10 at P42. **B** Red “+” on the fundus image demonstrate the measure points of ONL thickness at 500 µm from the optic nerve at superior, inferior, temporal, and nasal areas of the retina. **C** Representative retinal OCT images crossing optic nerve (yellow line in B) of control vehicle and ONL1204 injected rd10 at P28. **D** Quantification of the thickness of the ONL measured at 500 µm from the optic nerve head by OCT in control vehicle and ONL1204 injected rd10 mice at P28 (*n* = 21 mice), P35 (*n* = 16 mice), and P42 (*n* = 12 mice). Line graphs show paired value of both eyes from each individual mouse. One-tailed Wilcoxon test. INL inner nuclear layer, ONL outer nuclear layer.

The rd10 retina exhibits a nearly normal appearance during the initial two weeks of life, followed by a rapid loss of photoreceptors, with the peak of photoreceptor death occurring around P21 [44–46]. Typically, at 60 days of age, there are minimal nuclei left in the ONL, and ERG response is nearly nondetectable [46]. Given the rapid degeneration seen in rd10, we performed only one intravitreal injection of ONL1204 at P14. Caspase 8 activity, measured at P21, was significantly reduced in retinas of ONL1204 injected eyes as compared to control eyes (Fig. 4A). This was correlated with a significant decrease in the number of TUNEL-positive PR cells (Fig. 4B–D). This translated to increased ONL thickness in ONL1204-treated eyes, as compared to vehicle-treated control eyes assessed by retinal histology (Fig. 5A) as well as OCT measurement (Fig. 5B, C) at 28, 35 and 42 days of age.

**Fig. 6:**
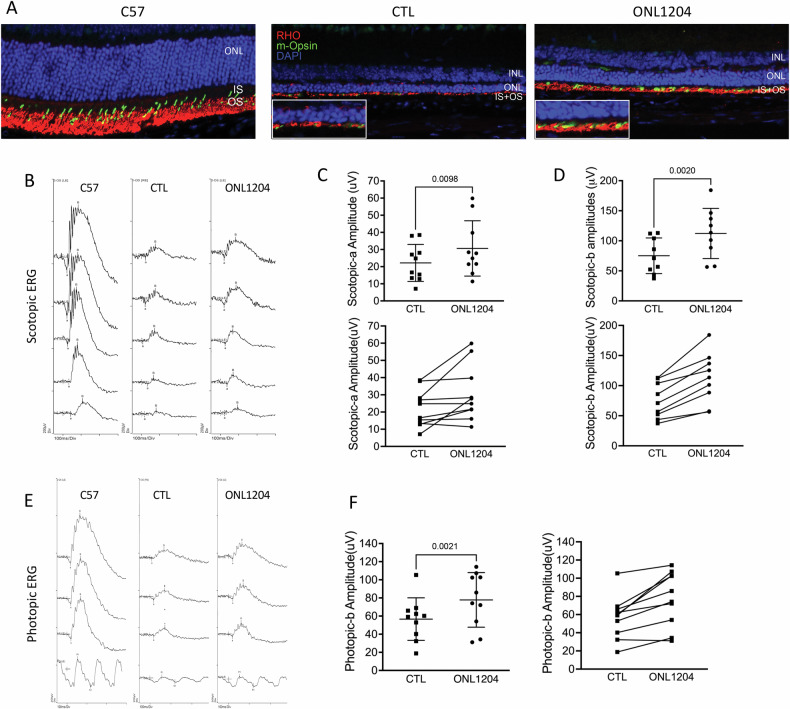
Improved retinal function in ONL1204 injected eyes in rd10 mice. **A** Representative immunostaining retinal images of C57, control vehicle and ONL1204 injected rd10 eyes at P42, stained with rhodopsin (RHO in red), m-opsin (green), and DAPI (blue). The white rectangular inserts are zoomed-in areas highlighting the staining in the inner and outer segments of the retinas. **B** Representative scotopic ERG traces of C57, and injected rd10 mice. **C** Quantification of amplitudes of scotopic a-wave, and (**D**) b-wave of ONL1204 and vehicle injected rd10 mice at P42 (36 cd s/m^2^). **E** Representative photopic ERG traces of C57, and injected rd10 mice. **F** Quantification of amplitudes of scotopic b-wave of ONL1204 and vehicle injected rd10 mice at P42 (100 cd s/m^2^). Line graphs show paired value of both eyes from each individual mouse. *n* = 10 mice; one-tailed Wilcoxon test; INL inner nuclear layer, ONL outer nuclear layer, IS inner segment, OS outer segment.

Immunostaining demonstrated an increased staining of rhodopsin and cone m-opsin within the retinas of ONL1204 injected eyes (Fig. 6A). Visual function measured by ERG confirmed that both scotopic (Fig. 6B–D) and photopic response (Fig. 6E, F) were significantly higher in ONL1204 injected eyes as compared to their respective control eyes. These findings collectively suggest that intravitreal administration of ONL1204 preserved photoreceptor viability and enhanced visual function in rd10 mice, mirroring the results observed in P23H mice.

**Fig. 7:**
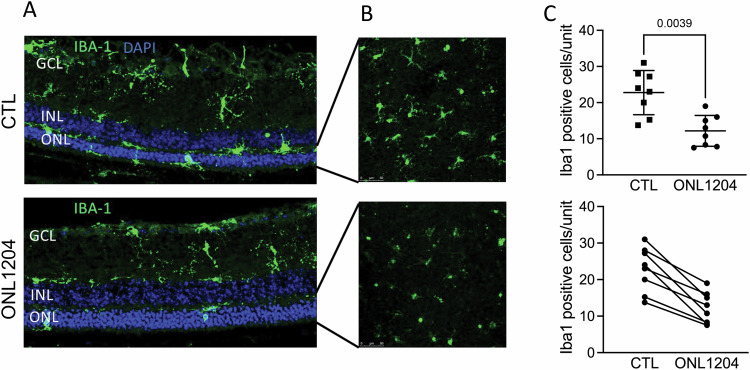
Injection of ONL1204 decreased the activation of immune cells in retina of rd10 mice. **A** Representative immunostaining images of retinal sections and (**B**) ONL from retinal whole mount of ONL1204 and control vehicle injected rd10 eyes at P21 stained with Iba-1 and DAPI. **C** Quantification of Iba1-positive cells in the ONL and subretinal space of control vehicle and ONL1204 injected rd10 eyes (*n* = 8 mice). Line graph shows paired value of both eyes from each individual mouse. One-tailed Wilcoxon test. GCL ganglion cell layer, INL inner nuclear layer, ONL outer nuclear layer.

Immunostaining for Iba1 on both 30 μm retinal sections (Fig. 7A), and retinal whole mounts (Fig. 7B) showed significantly decreased Iba1 positive immune cells in treated eyes (Fig. 7C). This is consistent with our results in P23H as well as in our Fas knockout in rd10, both of which showed reduced microglia/macrophage activation.

## Discussion

The Fas pathway is a key factor contributing to retinal cell death and the retinal inflammatory response in a variety of ocular diseases, including retinal detachment [7–10], AMD [11, 12], and glaucoma [6, 13]. In all these cases, even though different stressors can trigger the death of photoreceptor cells—such as the separation of the retina from the RPE (retinal detachment), oxidative stress (found in AMD), or increased pressure inside the eye (as in glaucoma)—there is a common pattern. Activation of the Fas pathway leads to propagation of cell death and an increase in inflammation within the retina. In animal studies of these diseases, blocking the activation of the Fas pathway significantly decreases the death of cells and reduces inflammation. This promising outcome has led to the initiation of three clinical trials in patients with these conditions, with the aim of investigating whether inhibiting the Fas pathway can protect their vision. (More information about these trials can be found on clinicaltrials.gov with the identifiers NCT03780972, NCT04744662, and NCT05160805).

In patients diagnosed with IRD, there is a critical need for treatments that can protect the structure and function of the retina. In the above-mentioned diseases, there is a heterogeneity in the stressor resulting in the activation of Fas. Similarly, in IRD there is a heterogeneity in the genetic mutations leading to disease. The diverse and numerous causative mutations make it exceptionally challenging to create specific treatments for each form of IRD. Our previous data demonstrate the upregulation and activation of the Fas pathway across various mutations [17]. Similar to other pathological conditions, Fas pathway activation in IRD coincides with the induction of inflammatory responses. Focusing on this shared core pathophysiological pathway underlying degeneration holds promise for mutation-independent therapeutic approaches, with the potential to extend the survival and enhance the functionality of retinal cells. In this study, we observed that ONL1204 had a positive impact in two separate models of IRD. Treatment with ONL1204 decreased Fas signaling, reduced the death of photoreceptor cells, lowered inflammation in the retina, and ultimately improved the overall functioning of the retina.

The inhibition of the Fas signaling pathway by ONL1204 leads to two principal outcomes. Firstly, it notably diminishes the activation of caspase 8. We have shown this to be a reliable surrogate measurement for reduced death pathway activation [7, 11, 12, 17] and this was corroborated in this study with reduced TUNEL-positive staining, increased retinal thickness, and improved function. The beneficial effect of ONL1204 was observed in both P23H and rd10 mouse models of IRD, consistent with our hypothesis that Fas activation is a shared pathophysiologic process leading to retinal degeneration. A secondary significant effect of diminished Fas activity in these models of IRD is the reduction in the activation of microglia and macrophages. This outcome is evidenced by a reduced presence of Iba1-positive cells in the retinas of both P23H and rd10 mice following ONL1204 injection. In mouse models of IRD, the activation of microglia and macrophages is well documented and is believed to play a role in the progression of retinal degeneration [47–51]. The ONL1204-associated decrease in microglia and macrophages activation may contribute to the protective effect, consistent with our previous work in other disease models [7–9, 11].

A significant limitation noted in our findings is that the protective effect of intravitreal ONL1204 injection when compared to control eyes, is only partial. Whereas the protective effect of Fas inhibition appears to be more pronounced in animal models of retinal detachment, AMD, and glaucoma, in the two models of IRD studied here, there is continued retinal degeneration, albeit at a reduced rate. Contrary to the diseases mentioned previously, the stressors in IRD are intrinsically derived, namely through the presence of a genetic mutations. This is as opposed to the presence of external stressors impacting otherwise normal photoreceptor cells, as observed in the other disease states. We suspect that in IRD there are additional, cell-autonomous contributors to cell death, and defining those pathways is an area of active investigation.

In conclusion, our findings indicate that intravitreal administration of ONL1204 in two mouse models of IRD leads to a decrease in Fas receptor activity. This reduction in activity subsequently results in the attenuation of cell death pathway activation and inflammation within the retina. This strategy focuses on a shared pathophysiological mechanism responsible for retinal cell death, without directly addressing the underlying genetic anomaly. While the use of ONL1204 for intravitreal injection presents a more viable route for clinical application compared to genetic manipulation of Fas inhibition, it is suggested that pharmacological inactivation of Fas might be most effective when integrated into a multi-therapy approach that targets various pathways contributing to retinal degeneration. Despite its partial efficacy, the benefits of pharmacologically reducing Fas activity could be substantial for patients suffering from these degenerative conditions. Further research is warranted to fully explore and validate this therapeutic potential.

## Data Availability

All data generated and analyzed in this study are presented in this published article. Primary data may be made available from the corresponding author upon reasonable request.
